# Dysregulation of leukocyte gene expression in women with medication-refractory depression versus healthy non-depressed controls

**DOI:** 10.1186/1471-244X-13-273

**Published:** 2013-10-21

**Authors:** Eli Iacob, Kathleen C Light, Scott C Tadler, Howard R Weeks, Andrea T White, Ronald W Hughen, Timothy A VanHaitsma, Lowry Bushnell, Alan R Light

**Affiliations:** 1 Department of Anesthesiology, University of Utah Health Sciences Center, Salt Lake City, UT, USA; 2 Department of Exercise and Sport Science, University of Utah, USA, Salt Lake City, UT, USA; 3 Department of Psychiatry, University of Utah, Salt Lake City, UT, USA; 4 Neuroscience Program, University of Utah Health Sciences Center, Salt Lake City, UT, USA

**Keywords:** Depression, Bipolar disorder, Medication-refractory, Females, qPCR, Gene expression, Immune, Purinergic receptor, Transient vanilloid receptor, Amyloid precursor protein, Oxytocin

## Abstract

**Background:**

Depressive Disorders (DD) are a great financial and social burden. Females display 70% higher rate of depression than males and more than 30% of these patients do not respond to conventional medications. Thus medication-refractory female patients are a large, under-served, group where new biological targets for intervention are greatly needed.

**Methods:**

We used real-time quantitative polymerase chain reaction (qPCR) to evaluate mRNA gene expression from peripheral blood leukocytes for 27 genes, including immune, HPA-axis, ion channels, and growth and transcription factors. Our sample included 23 females with medication refractory DD: 13 with major depressive disorder (MDD), 10 with bipolar disorder (BPD). Our comparison group was 19 healthy, non-depressed female controls. We examined differences in mRNA expression in DD vs. controls, in MDD vs. BPD, and in patients with greater vs. lesser depression severity.

**Results:**

DD patients showed increased expression for IL-10, IL-6, OXTR, P2RX7, P2RY1, and TRPV1. BPD patients showed increased APP, CREB1, NFKB1, NR3C1, and SPARC and decreased TNF expression. Depression severity was related to increased IL-10, P2RY1, P2RX1, and TRPV4 expression.

**Conclusions:**

These results support prior findings of dysregulation in immune genes, and provide preliminary evidence of dysregulation in purinergic and other ion channels in females with medication-refractory depression, and in transcription and growth factors in those with BPD. If replicated in future research examining protein levels as well as mRNA, these pathways could potentially be used to explore biological mechanisms of depression and to develop new drug targets.

## Background

For the many millions of patients who suffer from clinical depression worldwide, currently available antidepressant medications are effective in inducing sustained remission in less than half [1,2]. Patients who do not demonstrate symptom remission after two trials with antidepressants from different pharmacological classes are termed medication-resistant or medication-refractory. For these nonresponders to standard antidepressant treatments, there is urgent need to identify new potential targets for treatment, or to re-examine targets that were previously studied only in a heterogeneous pool of patients with depression. One approach to examining multiple potential molecular targets efficiently is to use leukocyte gene expression (mRNA) as biomarkers, which can be quantitatively compared to healthy controls or to other patient groups to indicate whether a specific target gene is over- or under-expressed.

Gender is frequently cited as a factor that may influence the course of depression and the response to antidepressant treatment. Women have a higher lifetime prevalence of depression than men [3] and the duration of treatment-induced remission is shorter for women than men [2]. Also, human postmortem studies of regional brain mRNA markers in depression have identified different patterns in females vs. males [4,5]. Consequently, one strategy to reduce heterogeneity when studying depressed patients employed by several recent studies is to focus exclusively on females [6-8].

Although there are numerous studies that report on gene expression differences in actively depressed patients with major depressive disorder (MDD) or bipolar disorder (BPD) compared to controls [9-11], few investigations have focused specifically on MDD and BPD patients with medication-refractory depression. Recently, Cattaneo et al. examined gene expression for the glucocorticoid receptor and related hypothalamic-pituitary-adrenal (HPA) axis genes, for pro- and anti-inflammatory cytokines, and for neuroplasticity and growth regulating factors in depressed patients prior to treatment with escitalopram or nortriptyline [12]. They reported that prior to starting treatment, higher expression of three pro-inflammatory cytokines was seen in those who subsequently were non-responders to treatment: TNF (formerly known as TNFα), interleukin (IL)-1β, and the macrophage inhibiting factor. Interestingly, compared to healthy controls, both treatment responders and non-responders also had elevated levels of these 3 cytokines and IL-6 but decreased levels of the glucocorticoid receptor NR3C1 and of three genes related to neuroplasticity prior to treatment. There was no treatment-related normalization for cytokines IL6 and TNF or for two of the neuroplasticity genes among the medication-refractory depressed patients, while medication-responsive patients did show normalization in all of these except TNF.

In the present study, we compared expression of 27 target genes in women with medication-refractory depression and healthy controls (see Table 1 for candidate genes and functional classes). We included genes previously implicated in clinical depression as well as more novel candidate genes previously studied in animal models of depression and anxiety but not in MDD or BPD patients. The target genes included 18 representing the three categories examined by Cattaneo et al. [12]. For the glucocorticoid pathway, we included the glucocorticoid and mineralocorticoid receptors, NR3C1 and NR3C2, respectively. Also related to the glucocorticoid pathway were the oxytocin prepropeptide and oxytocin receptor, OXT and OXTR, respectively. Oxytocin receptor polymorphisms have been associated with depression and anxiety [13-15], and oxytocinergic activity has been shown to be dysregulated in women with both unipolar and bipolar depression [16-18], and it has been suggested as a potential therapeutic target for mental disorders [19,20]. For cytokines, we included pro-inflammatory TNF, LTA, and IL-6, as well as anti-inflammatory IL-10. For neuroplasticity and growth regulation, we included 10 genes: APP, CREB1, DBI, NFKB1, NRG1, PPARA, SIRT1, SPARC, STAT5A, and VEGFA. We also included another category of target mRNAs that have not yet been examined on leukocytes of patients with refractory depression but have been reported to be dysregulated in patients with Chronic Fatigue Syndrome (CFS) and Fibromylagia (FM), many of whom had depressive symptoms [21] and are implicated in animal models of anxiety and depression [22-24]. These are ion channel receptors responding to ATP or ADP and other metabolites, and included acid sensing ion channels ASIC1 and ASIC3, and purinergic receptors P2RX1, P2RX4, P2RX7, P2RY1 and P2RY2. Also included were two transient vanilloid receptors, TRPV1 (the capsaicin receptor) and TRPV4 (another heat receptor). Smolin et al. previously reported that ASIC1 expression was dysregulated in postmortem brain tissue of patients with psychiatric disorders [25]. Perreira et al. have recently reported that non-selective P2 receptor antagonists, more specifically P2X antagonists, have antidepressant effects in mice [26]. Several studies have also identified single nucleotide polymorphisms (SNPs) of the P2RX7 receptor to be significantly associated with both MDD and BPD [27], and this purinergic receptor was under-expressed in patients with post-traumatic stress disorder, including many with comorbid depression [28]. Our goals were: 1) to extend prior findings indicating that neuroplasticity, cytokine, and glucocorticoid mRNAs (including those mRNAs specific for oxytocinergic activity) were differentially expressed on leukocytes in women with medication-refractory depression vs. controls, and in patients with BPD vs. MDD; 2) to provide an initial examination of possible differential leukocyte expression of ion channel receptors in these same patient groups, and 3) to identify any of these mRNAs that were correlated with depression symptom severity, defined by self-report scores on the Quick Inventory of Depressive Symptomatology (QIDS).

**Table 1:** Candidate genes for mRNA gene expression in DD

**Pathways**	**Classes**	**Genes**
HPA	Glucocorticoid	NR3C1
	Mineralocorticoid	NR3C2
	Oxytocin	OXT
	Oxytocin receptor	OXTR
Immune response and inflammation	Cytokines	IL-10, IL-6, TNF, LTA
Transcription factors	Gene expression factors	CREB1, SIRT1, STAT5A, PPARA, NFκB1
Neuronal health and signaling	Growth factors	NRG1, VEGFA
	Matrix associated	APP, SPARC
	Modulator	DBI
Ion channels	Purinergic	P2RX1, P2RX4, P2RX7, P2RY1,
	Acid sensing	P2RY2
	Transient	ASIC1, ASIC3
	Vanilloid	TRPV1, TRPV4

## Methods

### Subjects

All participants were non-pregnant and 20–72 years old. All subjects gave written consent, and protocols were reviewed and approved by the Institutional Review Board (University of Utah). The Depression Disorders (DD) group included 23 females in an active state of moderate to severe depression who were receiving a consult for Electroconvulsive Therapy (ECT) having had unsatisfactory response to three or more different antidepressant medication regimens. Diagnosis of MDD (n=13, ages 26–60) or BPD (n=10, ages 26–72) was made by psychiatrists HW and LB according to the criteria in the *Diagnostic and Statistical Manual of Mental Disorders, Fourth Edition* (DSM-IV) [29]. Diagnosis, current medications, inpatient/outpatient status, and medical comorbidities for each individual patient, are included as Additional file 1: Table S1.

Clinical assessment of depression severity (Table 2) was carried out using the Hamilton Rating Scale of Depression (HRSD)-24 score, a structured clinical interview, scored by the psychiatrist and other trained staff during the initial clinical visit prior to any change in treatment [30,31]. Depression severity was also assessed using the Quick Inventory of Depressive Symptomatology (QIDS) Self Report either at the initial visit or another clinical visit prior to any new treatment (Table 2). The QIDS self report has been found to have concurrent validity with the clinician based QIDS and the Hamilton Rating Scale of Depression [32]. The control group (CON), which included 19 individuals, 23–68 years old, also completed the QIDS, and provided demographic and medical history information including history of depression and other mental disorders. We excluded any controls that had been previously diagnosed with depression, were currently taking antidepressants, or had a QIDS score in the mild or greater depression range. Evaluation of medication-responsive subjects is left for a future study.

**Table 2:** Demographic summary for CON and DD

	**CON n=19**	**MDD n=13**	**BPD n=13**	**DD (combined) n=23**
Age (years)	46.6±2.9	44.6±3.3	48.1±4.6	46.1±2.7
HRSD		36.6±3.7	33.8±3.0	35.4±2.4
QIDS^A^	3.32±0.6	19.67±1.5	19.38±1.3	19.55±1.0
No medication	17	0	0	0
Antidepressants^B^	0	13	6	19
Anticonvulsants (N/S/C)^C^	(17/0/2)	(3/2/8)	(3/1/6)	(6/3/14)
Antipsychotic	0	5	6	11

^A^QIDS was significantly higher in MDD (*p* <0.001), BPD (*p* <0.001), and DD (*p* <0.001) relative to CON.

^B^Antidepressant use has higher in MDD vs. BPD χ^2^ 4.749 (*p* =0.01) and vs. CON (*p* <0.01).

^C^Anticonvulsant (N = not on, S = stopped in anticipation of ECT, C = currently on).

Concurrent with behavioral assessment, blood samples were obtained from each participant based on patient and psychiatrist availability between 10 am-3 pm. Patients and controls were matched for age, with no significant differences between groups (t = 0.1135, *p* = 0.91). Antidepressant use was significantly higher in patients with MDD vs. BPD (χ^2^ = 6.29, *p* = 0.03). There were no differences between MDD and BPD for anticonvulsant use (χ^2^ = 0.232, *p* = 0.891) or antipsychotics (χ^2^ = 0.306, *p* = 0.580).

### mRNA extraction and analysis

All blood processing was performed by personnel blinded to the subjects’ group. Blood was collected in EDTA tubes and kept on ice. Within 15 minutes after collection, the blood was centrifuged at 3200 rpm (1315 × g- Clay Adams Compact II Centrifuge) for 12 minutes, plasma was removed, and the white layer was carefully collected in RLT+β-ME (Qiagen, Valencia, CA), then quickly frozen using a methanol-dry ice slurry, and stored at −80°C. RNA was extracted using RNeasy mini kits (Qiagen, Valencia, CA), and treated with RNase-free DNase-I (Qiagen, Valencia, CA). Immediately following extraction, RNA was converted to a cDNA library using the ABI High Capacity cDNA Archive Kit (Applied Biosystems/Life Technologies, Inc., Foster City, CA), followed by treatment with RNase-H (Epicentre Biotechnologies, Madison, WI). The cDNA samples were stored at -20°C until further use. RNA integrity was assessed with a Bioanalyzer, and consistently found to have RIN values (RNA integrity numbers) greater than 9. The cycle counts for the control gene, TF2B, averaged 22.81 ± 0.16 (SE) for DD patients and 23.21 ± 0.19 for CON subjects (t = 1.63, *p* = 0.11). We selected TF2B as the preferred control gene and have verified that TF2B qPCR counts do not change in freshly harvested separated human leukocytes under a variety of conditions, when RNA is processed as described here.

The cDNA libraries were analyzed using the ABI quantitative, real-time PCR system on the ABI Prism 7900 Sequence Detection System (SDS) 2.4.1 (Applied Biosystems, Inc., Foster City, CA), using ABI TaqMan Master Mix (Applied Biosystems, Inc., Foster City, CA). Master mix/primer plus primer probe solutions and template solutions were separately loaded onto 96 well source plates. Then 384 well plates were robotically loaded and mixed from the 96 well plates. Each targeted gene was examined in duplicate, with TF2B standards being run in quadruplicate. Additional control samples containing no template were also run. Primer-probes for the 27 candidate genes were obtained from TaqMan Gene Expression Assays (Applied Biosystems, Inc., Foster City, CA) and are listed in Additional file 2: Table S2. qPCR data was processed using the SDS program, with count values for genes computed in the curve log-linear range using a standard 0.2 threshold. Gene expression amounts were determined using the 2^-ΔT^ method, where ΔT is the count difference of the candidate gene from TF2B.

### Statistical analysis

All statistical tests were made using STATA ver. 12 statistical software. Distributions of the mRNA data showed considerable skew. To address this normality, we performed all parametric tests using log transformed data. To ensure that our data were not biased by outliers, we also repeated all tests with non-parametric Wilcoxon rank-sum tests. First, we used General Linear Models (GLM) to identify any differences in gene expression between all DD patients vs. nondepressed CON; age was found to be a significant covariate for only 2 genes, ASIC1 and LTA (*p* < 0.025), and so was retained in those final models but dropped for the other 28 genes. Antidepressant and antipsychotic medication use could not be included as a potential covariate in these analyses because none of the healthy controls were taking these medications. Cohen’s d effect sizes were calculated for all between group comparisons.

Second, using the mRNA data from the DD patients only, we used multiple regression analysis to examine the orthogonal effects of 5 factors: 1) BPD vs. MDD diagnosis, 2) depression severity (defined first by QIDS score, and then repeated using HRSD score), 3) use of antidepressant and antipsychotic medications (coded as 0=none, 1= SSRI/SNRI/NRI antidepressants only, 2=antipsychotic only, 3=antidepressants + antipsychotic), 4) use of anticonvulsant (0=none or stopped, 1=anticonvulsant), and 5) age. In this second set of analyses, we thus could determine whether there were differences in gene expression between BPD and MDD patients after controlling for effects of depression severity and vice versa. We also could assess whether either the diagnosis or severity factors remained significant after controlling for medications and age. Alpha level for all analyses was set at *p* < 0.05. η^2^ effect sizes were computed for regression models.

## Results

### Differentially expressed genes in DD compared to CON

Our 27 candidate gene panel included genes from diverse functional classes; however, only genes from the immune, neuronal health and ion channel classes differed between actively depressed patients and controls. We observed that 6 genes were significantly increased in DD relative to CON [APP (F 1,40 =4.37, *p* = 0.043, Wilcoxon rank sum on non-transformed data *p* = 0.029), IL-10 (F 1,40 =4.29, *p* = 0.045, *p* = 0.039), IL-6 (F 1,40 = 5.18, *p* = 0.028, *p* = 0.045), OXTR (F 1,40 = 5.27, *p* = 0.023, *p* = 0.037), P2RX7 (F 1,40 = 4.40, *p* = 0.042, *p* = 0.050), P2RY1 (F 1,40 = 6.42, *p* = 0.015, *p* = 0.015), and TRPV1 (F 1,40 = 6.03, *p* = 0.019, *p* = 0.010)] (see Table 3). Age was not a significant or marginally significant covariate for any of these genes. None of our 27 candidate genes showed significantly lower expression in DD versus CON.

**Table 3:** Gene expression (mean ± SE) for genes differing between all patients with Depressive Disorders (DD) versus Controls (CON)

**GENE**	**CON (n=19)**	**DD (n=23)**	**p value**^**A**^	**p value**^**B**^	**Cohen’s d**^**C**^
			**Log transformed**	**Raw data**	
**APP**	5.65E-01 ± 3.12E-02	6.70E-01 ± 3.59E-02	0.043	0.029	0.661
**IL-10**	3.86E-03 ± 3.66E-04	5.27E-03 ± 5.33E-04	0.045	0.039	0.655
**IL-6**	2.02E-03 ± 2.79E-04	2.94E-03 ± 3.45E-04	0.028	0.045	0.719
**OXTR**	2.94E-03 ± 3.00E-04	4.75E-03 ± 6.95E-04	0.079	0.037	0.569
**P2RX7**	4.64E-02 ± 3.22E-03	5.84E-02 ± 4.89E-03	0.042	0.050	0.663
**P2RY1**	7.48E-02 ± 3.89E-03	9.45E-02 ± 5.82E-03	0.015	0.015	0.801
**TRPV1**	1.15E-02 ± 6.36E-04	1.35E-02 ± 5.07E-04	0.019	0.010	0.777

Raw data is displayed in the table. Expressed in scientific notation units where E+00 = x1, E-01 = x0.1, E-02 = x0.01, etc. Data were log transformed prior to analysis.

^A^Differences between DD vs. CON examined using log transformed data and Student’s t-test.

^B^Differences between DD vs. CON examined using non-transformed data and nonparametric Wilcoxon Rank Sum test.

All comparisons had 40 degrees of freedom and F values ranging from 4.29 – 6.42.

^C^Cohen d effect sizes were all moderate to large and are reported with DD as the reference group.

### Effects of medication use and age in the depressed patient sample

In our 5-factor regression model, use of antidepressant and antipsychotic medication was significantly related to higher expression of NR3C2 (adjusted t = 2.60, *p* = 0.026) and ASIC1 (adjusted t = 2.39, *p* = 0.029). However, this medication factor was also marginally related to increased or decreased expression of 3 other genes: OXTR (adjusted t = −1.91, *p* = 0.073), SIRT1 (adjusted t = 1.77, *p* = 0.094), ASIC3 (adjusted t = 2.01, *p* = 0.06). Given our relatively small sample size, which limits statistical power, these trends suggest that controlling for use of these medications is potentially important.

Anticonvulsant use was likewise marginally related to increased VEGF (adjusted t = 1.88, *p* = 0.078), APP (adjusted t = 2.01, *p* = 0.061), and ASIC3 (adjusted t = 1.96, *p* = 0.067). Thus, we felt that it was important to retain both medication factors in our final models, and thus to examine effects of other factors after adjustment for use of these different types of medications.

Age was significantly related to lower expression of 2 genes, NFKB1 (adjusted t = −2.98, *p* = 0.008) and LTA (adjusted t = −3.11, *p* = 0.006).

### Effects of BPD versus MDD diagnosis

After controlling for medications, age and depression severity, currently depressed patients with a diagnosis of BPD showed higher expression of 5 genes and lower expression of 1 gene than patients with a diagnosis of MDD (see Table 4). These over-expressed genes included 3 transcription factors: CREB1 (adjusted t = −2.50, *p* = 0.023), NFKB1 (adjusted t = −2.30, *p* = 0.035), and NR3C1 (adjusted t = −2.18, *p* = 0.044. They also included 2 matrix-associated genes, APP (adjusted t = −3.13, *p* = 0.006) and SPARC (adjusted t = −3.37, *p* = 0.004). Patients with BPD displayed decreased expression in one immune gene, TNF (adjusted t = 2.32, *p* = 0.033).

**Table 4:** Gene expression (mean ± SE) for genes differing between BPD and MDD subgroups

**GENE**	**BPD (n=10)**	**MDD (n=13)**	**p value**^**A**^	**η2**
**APP**	7.63E-01± 4.80E-02	5.98E-01 ± 4.33E-02	0.006	0.303
**CREB1**	7.40E-01 ± 4.75E-02	6.39E-01 ± 2.15E-02	0.028	0.210
**NFKB1**	5.81E-01 ± 2.42E-02	5.29E-01 ± 1.99E-02	0.038	0.179
**NR3C1**	3.27E-01 ± 2.81E-02	2.78E-01 ± 9.51E-03	0.043	0.191
**SPARC**	3.20E+00 ± 2.72E-01	2.29E+00 ± 1.80E-01	0.003	0.300
**TNF**	1.36E-01 ± 5.99E-03	1.68E-01 ± 1.14E-02	0.032	0.200

Raw data is displayed in the table. Expressed in scientific notation units where E+00 = x1, E-01 = x0.1, E-02 = x0.01, etc. Data were log transformed prior to analysis.

^A^BPD vs. MDD using 5 factor regression model with inclusion of age, QIDS-SR, anticonvulsant use, and antidepressant/antipsychotic use. Model has 23 patients, 5 variables, results in 17 degrees of freedom.

p value is for effect of Diagnostic subgroup after adjusting for the effects of the other 4 factors.

Effect sizes using η2 for where diagnostic subgroup was significant in the regression model were consistently large (>0.14).

### Effects of depression severity

After controlling for other factors listed above, depression severity (defined by QIDS score) was significantly related to expression of 4 genes (Table 5). These included increased expression of 3 ion channel genes: P2RY1 (adjusted t = 3.12, *p* = 0.006), P2RX1 (adjusted t = 2.86, *p* = 0.011) and TRPV4 (adjusted t = 2.29, *p* = 0.035). Severity was also associated with increased expression of IL-10 (adjusted t = 2.28, *p* = 0.036). When other non-significant components of the models were dropped, all of these effects of depression severity on these 4 mRNAs were significant (p<0.03). In a separate 5-factor regression model using the clinical HRSD for depression severity, severity was associated with increased expression of P2RX1 (adjusted t = 2.44, *p* = 0.026) and P2RY1 (adjusted t = 2.10, *p* = 0.051), but not TRPV4 (adjusted t = −0.14, *p* = 0.893) or IL10 (adjusted t = −0.35, *p* = 0.734).

**Table 5:** Significant associations of depression severity and gene expression for HRSD and QIDS controlling for BPD vs. MDD diagnosis, age, and medication use

**GENE**	***QIDS *** **t**	**p value**	**η2**	***HRSD *** **t**	**p value**	**η2**
**P2RX1**	2.86	0.011	0.296	2.44	0.026	0.236
**P2RY1**	3.12	0.006	0.333	2.10	0.051	0.189
**TRPV4**	2.29	0.035	0.234	−0.14	0.893	0.001
**IL10**	2.28	0.036	0.227	−0.35	0.734	0.007

Depression severity assessed by QIDS or HRSD using 5 factor regression model with inclusion of diagnosis, age, anticonvulsant use, and antidepressant/antipsychotic use. Model has 23 patients, 5 variables, results in 17 degrees of freedom.

p value is for effect of depression severity after adjusting for the effects of the other 4 factors.

Effect sizes using η2 for where depression severity was significant in the regression model were large (>0.14) for the 4 genes using QIDS and for P2RX1 and P2RY1 using HRSD.

## Discussion

New biological targets for treatment are urgently needed for patients with depressive disorders (both unipolar and bipolar), and the population where this need is most critical are those with medication-refractory depression. Identifying more of the diverse neuroimmune pathways that may be dysregulated in these disorders provides a first step in this process. As a screen for possible dysregulation, we looked for increased or decreased gene expression using qPCR from peripheral leukocytes in patients with medication-refractory depression vs. healthy controls with no history of depression. Specifically, we examined genes representing several biological pathways including glucocorticoid receptors, immune function, growth and transcription factors, and purinergic, transient vanilloid and acid sensing ion channel receptors. We restricted our sample to females to eliminate gender as a potential source of heterogeneity; however, we included depressed patients with both MDD and BPD diagnoses and secondarily examined differences in gene expression between these depression subgroups while controlling for medication use and depression severity. Finally, we examined relationships of increased or decreased expression to greater symptom severity, after controlling for several potential confounding factors, including medications.

Using this approach, our observations reconfirmed dysregulation in two immune genes previously implicated in depression: the cytokines IL-10 and IL-6 [33-35]. Our findings also provided initial evidence of possible dysregulation in 3 relatively novel targets for depression: ion channels TRPV1, P2RX7 and P2RY1. All of the above genes showed increased expression in DD patients compared to healthy controls; none of our targeted genes showed decreased expression among patients. With the DD sample, greater depression severity (whether defined by QIDS or by HRSD scores) was associated with higher expression of ion channels P2RX1 and P2RY1. In addition, greater depression was associated with higher expression of IL-10 and TRPV4 using QIDS as the severity measure. These associations further strengthen the interpretation that IL-10 and ion channels are prominent targets for intervention development in medication-refractory depression.

Further analyses suggested possible differences between BPD and MDD subgroups after controlling the effects of medication use and depression severity. These genes included 2 genes in the HPA-immune axis: the glucocorticoid receptor NR3C1 and the cytokine TNF which were upregulated and downregulated in BPD, respectively. They also included upregulation of 2 transcription factors with widespread effects on regulation of other neuroimmune genes: CREB1 and NFKB1. In addition, they included upregulation of 2 matrix-associated growth factors, SPARC and APP. Although APP was also seen to be up-regulated in all DD patients versus healthy controls, examination of the group means (Tables 3 and 4) showed that this effect was primarily due to the BPD group.

### Differentially expressed genes and their possible functional significance

Gene expression dysregulation may be indicative of imbalance or dysfunction in these biological pathways and therefore, implicate potential drug targets. Clinical studies previously have examined the effects of antidepressant and antipsychotic medications on biomarkers of inflammation [36-38], glucocorticoid receptors [39], glutamate [40], and neuropeptides [19], among others. Immune and HPA axis activity have thus been the most common targets of antidepressant intervention [41-43] other than monoamine systems involving serotonin and norepinephrine which are primary targets of SSRI/SNRI/NRI and tricylic medications.

Consistent with previous studies on mRNA in blood of patients with depression, both medication responders and non-responders, we found upregulation in cytokines IL-6 and IL-10 in females with medication-refractory depression [9,12,44]. Protein elevations of both cytokines in serum and CSF are a consistent finding in depression [41,45,46], with some studies suggesting that IL-6 is specifically associated with medication-refractory depression, and that IL-10 levels decrease following successful treatments [33-35]. Because we found that IL-10 levels were positively correlated with depression severity, it will be important for future studies to examine gene expression levels following symptom remission.

Our finding of increased peripheral OXTR mRNA in DD patients may reflect dysregulation in oxytocinergic signaling. Also, the trend shown in the present study for the association between the use of antidepressant medications and lower OXTR expression suggests that such decreases may contribute to beneficial effects of these medications. Oxytocin is a neuropeptide that is synthesized and released by the hypothalamus, and has been implicated in many biological functions, including social bonding, anxiety, pleasure-seeking, appetite, and stress response, all of which can be disrupted in depression [47]. Most studies suggest that low plasma and CSF levels of oxytocin are associated with depressive behaviors and increased sensitivity to stressors, and that such differences may be specific to depressed females rather than to males [18,47,48]. Furthermore, subjects with depression display greater fluctuations in oxytocin in response to stressful mental tasks, suggesting dysregulation [16,49]. In addition, polymorphisms in the OXTR can result in decreased receptor expression, symptoms of depression and anxiety, and sensitivity to stressors [13-15]. Since intranasal oxytocin may have beneficial effects on depressive symptoms and anxiety, patients displaying oxytocin pathway mRNA dysregulation could benefit from such a targeted treatment [17].

Of the growth factors that we examined, we observed that female patients with depression displayed elevated levels of amyloid precursor protein (APP) mRNA compared to healthy non-depressed controls. Subsequent analysis found that patients with BPD had higher levels of APP compared to MDD suggesting increases vs. CON are primarily due to the BPD subgroup. APP (most notable for its association with Alzheimer's Disease) plays multiple roles, for instance that of a neuronal growth factor and in transcriptional regulation, with complicated post-translational processing resulting in multiple cleaved peptides. Therefore, the increased APP gene expression that we observed may be due to compensation for low peripheral levels of peptides, or to non-functional peptides. For example, alterations in CSF APP peptides have been observed in patients with BPD, including decreased levels of sAPP-α and sAPP-β in Bipolar type I and changes in ratios for other Aβ peptide products in Bipolar type II [50]. Decreased levels of Aβ40 and Aβ42 have also been observed in the CSF and serum of depressed patients with levels correlating to depression severity [51-53], though both increased and decreased levels of Aβ42 have been found in human plasma [54,55]. Given the complex post-transcriptional processing of APP, future studies should combine mRNA and protein analysis to determine the relationship between APP transcript levels and peptide products, and if levels differ between medication-refractory and medication-responsive patients.

Our gene expression panel also consisted of several ligand-gated channel families important in detecting ATP and other metabolites that may be related to pain and fatigue, frequent symptoms in DD. Of the 9 ion channel receptors that we examined, P2RX7, TRPV1, and P2RY1 were upregulated in female patients during a depressive episode, and P2RY1 expression was positively related to depression severity along with 2 other ion channel receptors, TRPV4 and P2RX1. Our clinical findings in regard to P2RY1, P2RX1, TRPV1, and TRPV4 mRNAs are the first such observations in depressed humans; however they are consistent with a number of preclinical and genetic studies. Modulation of TRPV1 and P2RY1 receptors in animals has effects on depression and anxiety tasks [23,24,56]. Furthermore, our research group previously found that following a moderate exercise task, when their fatigue and pain symptoms worsen, patients with CFS show increased expression of ion channels, with expression levels positively correlated with pain and fatigue symptoms [21,57]. While the role of peripheral P2RY1 receptor function in human depression is unknown, it is promising that we observed gene expression increases in DD that were also positively correlated to depression severity, suggesting either that increased expression may be involved in symptom presentation or that it is compensatory for receptor dysfunction.

Some prior studies have suggested that certain polymorphisms in P2RX7 may predispose individuals to MDD, although these findings have been disputed by others [58-60]. Our results of increased P2RX7 mRNA expression in patients with medication refractory DD are contrary to those reported by Zhang et al., who found decreased peripheral expression of P2RX7 in psychiatric patients (primarily female) with post-traumatic stress disorder or MDD, and similar mRNA decreases in postmortem tissue from suicide victims in a meta analysis [28]. Our samples are clearly different in several characteristics, but it is also possible that our increased peripheral receptor mRNA reflects secondary compensatory increases to diminished central activity in this pathway. Still, our observations as those of Zhang et al. are consistent in indicating dysregulation in this purinergic pathway in DD.

Although purinergic P2X and P2Y, acid-sensing ASIC, and transient vanilloid TRPV ion channels have been the target for the treatment of multiple other disorders, including thrombosis, cardiac arrhythmias, and neuropathic pain [61,62], these pathways have not yet been examined for antidepressant effects in patients with MDD or BPD. Given the numerous animal studies implicating ion channels including P2X, P2Y, and TRPV receptor function in anxiety- and depression-like states, and our studies showing dysregulation in these ion channel receptors in refractory depression, further investigation of drugs or other interventions targeting ion channels for depressive symptoms may be warranted.

Though still in their infancy, gene expression studies may one day help to identify individuals that would most benefit from drugs targeting non-monoamine pathways.

### Effects of medications, clinical diagnosis, and depression severity on gene expression

In order to explore possible confounders in our DD sample, we included medication use, depression severity, and DD diagnosis in a five-factor regression model. Medications have been shown to affect immune function and alter gene expression [10,37,63-66]. We found significant effects of medication for NR3C2, ASIC1, and APP with ASIC3, OXTR, and SIRT1 showing marginal effects. Importantly, with the exception of APP and OXTR, none of these genes were identified in either our DD vs. CON or BPD vs. MDD analyses. Decreased expression of OXTR with medication use suggests that medications may have minimized rather than contributed to the higher expression in DD vs. CON. Conversely, anticonvulsant use was associated with higher expression of APP and therefore medication use could have contributed to elevated levels in DD vs. CON. Future work should continue to consider the effects of medication use on gene expression and how it may accentuate or mask group differences.

In our sample we focused on patients that were currently in a depressive state. This included patients with BPD and MDD diagnosis. We identified several genes that showed group differences after controlling for concurrent psychotropic medication use, age, and depression severity. These included BPD elevations in transcription factors CREB1, NR3C1, and NFKB1, matrix-associated genes APP and SPARC, and downregulation of proinflammatory cytokine TNF. Of these genes, only APP was also found to be confounded by anticonvulsant use. However, robust differences persisted even after controlling for medication use. The same genes displayed differences between BPD and MDD when using HRSD. These are promising results that suggest possible biomarker differences between these diagnostic groups, but future replication studies are necessary.

Finally, we examined associations between depression severity and gene expression using both the clinical interview HRSD and QIDS self report scores. Both the QIDS and HRSD displayed positive associations for depression and gene expression for ion channels P2RX1 and P2RY1, further strengthening the possible relationship of ion channels and depression. In addition, QIDS had a positive association for another ion channel, TRPV4, and for the cytokine IL10, which was also identified as up-regulated in DD vs. CON in the primary analysis. HRSD score was not associated with these latter two genes (Table 5). Although QIDS and HRSD scores were strongly related to each other (R^2^ = 0.586, *p* = 0.003), as is always true, they were not identical. Future mRNA studies may benefit by including both assessment methods.

### Limitations

There are several noteworthy limitations to this study. Because we examined female subjects only, it is not possible to conclude whether the observed gene expression differences would extend to depressed males or represent true gender-specific differences. Secondly, in this study we did not include control subjects taking antidepressants or antipsychotics. For example, since there were only 4 DD patients that were not taking antidepressants, it is possible that gene expression differences were influenced by antidepressant use. Medication-responsive control subjects likely represent a distinct group from healthy non-depressed controls and must be treated differently. In our regression analysis of depressed patients only, we did find that medication use led to differences in expression when controlling for age, depression severity, and diagnosis, including increased expression of ASIC receptors, transcription factor NR3C2, and growth factor APP. It is critical for future studies to examine gene expression in light of medication use. Thirdly, our primary analysis combined patients with BPD and MDD into a single group when comparing to non-depressed controls. This was done partially because all of our patients were currently in a depressed state. Further regression analysis controlling for depression severity, age, and medication, revealed several potential differences based on diagnosis. These results are promising in the search for potential diagnostic biomarkers, but our small sample size warrant future studies where BPD and MDD groups with adequate power are also examined separately compared to controls. Finally, it is important to note that depression remains enigmatic and difficult to treat, partially because of the diversity of disease onset, symptom presentation, comorbidities, and disease evolution. Current studies are under way in patients with depression to examine gene expression changes following moderate exercise tasks, found to differentiate patients with CFS and FM from controls. Similar to the study by Cattaneo et al. [12], future research should compare gene expression over time for treatment-resistant and treatment-responsive patients in prospective studies before and after symptom remission. By amassing a panel of differentially expressed genes, researchers could continue to identify novel biological mechanisms contributing to depression and potentially pave the way to new drug targets and personalized medicine.

## Conclusions

The principal finding of this study is that women with medication-refractory depression display increased expression of genes from diverse biological pathways, including the immune genes IL10 and IL6, the oxytocin receptor OXTR, the growth factor APP, and the metabolite receptors P2RY1, P2RX7, and TRPV1. Furthermore, differences between MDD and BPD were seen for matrix-associated growth factors APP and SPARC, immune gene TNF, and transcription factors CREB1, NFKB1, and NR3C1 when controlling for depression severity, age, and medication use. Finally, two additional ion channel genes, P2RX1 and TRPV4, as well as two of the genes from our primary analysis, IL-10 and P2RY1, were related to depression severity as assessed by the QIDS and/or HRSD. Future replication studies, particularly those measuring protein levels as well as mRNA, are still needed to strengthen the case for development of new targeted treatments.

## Abbreviations

mRNA: Messenger ribonucleic acid; qPCR: Real-time quantitative Polymerase Chain Reaction; MDD: Major depressive disorder; BPD: Bipolar disorder; DD: Depressive disorder group; CON: Control group; CFS: Chronic fatigue syndrome; FM: Fibromyalgia; QIDS: Quick Inventory of Depressive Symptomatology; ECT: Electroconvulsive therapy; GLM: General linear model; HPA-axis: Hypothalamic-pituitary-adrenal axis; CSF: Cerebral spinal fluid; MR: Mineralocorticoid receptor; GR: Glucocorticoid receptor; NR3C: Nuclear receptor subfamily 3, group C; OXT: Oxytocin; OXTR: Oxytocin receptor; IL-10: Interleukin-10; IL-6: Interleukin-6; TNF: Tumor necrosis factor α; LTA: Lymphotoxin α; P2R: Purinergic Type 2 receptors; TRPV: Transient Receptor Potential Vanilloid; ASIC: Acid Sensing Ion Channel; HCN2: Hyperpolarization activated cyclic nucleotide-gated potassium channel 2; CREB1: cAMP responsive element binding protein 1; SIRT1: Sirtuin 1; STAT5A: Signal transducer and activator of transcription 5A; PPARA: Peroxisome proliferator-activated receptor alpha; NFKB1: Nuclear factor kappa in B cells; NRG1: Neuregulin 1; VEGFA: Vascular endothelial growth factor A; APP: Amyloid precursor protein; SPARC: Secreted protein, acidic, cysteine-rich; DBI: Diazepam binding inhibitor.

## Competing interests

The authors declare that they have no competing interests.

## Authors’ contributions

EI participated in study design, coordination and acquisition of data, screened patients and controls, carried out gene expression analysis, performed the statistical analyses, and drafted the manuscript. KL participated in study design, wrote grant proposals providing study support, helped with statistical analyses, and co-wrote the manuscript. HW and LB helped in recruitment of patients. ST helped with study design and with writing of grant proposals providing study support. AW screened patients and controls, and performed blood draws. RW and TV screened controls and processed blood for qPCR gene expression. AL participated in study design, helped with gene expression analysis and statistical analysis, and co-wrote the manuscript. All authors read and approved the final manuscript.

## Pre-publication history

The pre-publication history for this paper can be accessed here:

http://www.biomedcentral.com/1471-244X/13/273/prepub

## Supplementary Material

Additional file 1: Table S1 Patient demographics. Click here for file

Additional file 2: Table S2 List of ABI Primers (Taqman Assays) used for qPCR. Click here for file
