# Supplementary material for: Dysregulation of leukocyte gene expression in women with medication-refractory depression versus healthy non-depressed controls
Source: BMC Psychiatry. 2013 Oct 21;13:273. doi: 10.1186/1471-244X-13-273 (PMC4015603; doi:10.1186/1471-244X-13-273)
Supplement: Additional file 1: Table S1 — Patient demographics. [file 1471-244X-13-273-S1.doc]

Supplemental Table 1: Patient demographics

| **Case** | **Gender** | **Age (yrs)** | **Diagnosis** | **Inpatient/**  **Outpatient** | **CNS medication^A^** | **HRSD** | **QIDS-SR** |
| --- | --- | --- | --- | --- | --- | --- | --- |
| **501** | F | 47 | BPD I | Outpt | celexa, saphris | 41 | 19 |
| **503** | F | 40 | MDD, anxiety, panic disorder | Inpt | wellbutrin, celexa, zyprexa, lithium, diazepam | 53 | 27 |
| **505** | F | 36 | MDD | Inpt | wellbutrin, cymbalta, abilify | 44 | 22 |
| **506** | F | 56 | MDD | Outpt | wellbutrin, seroquel, xanax | 37 | 25 |
| **507** | F | 60 | MDD | Outpt | pristiq, pilocarpine, xanax | 40 | 25 |
| **508** | F | 54 | MDD, Anxiety | Outpt | wellbutrin, lithium, risperdal, klonopin, Xanax | 35 | 17 |
| **509** | F | 57 | MDD | Inpt | viibryd, pristiq, wellbutrin, topamax | 46 | 22 |
| **511** | F | 39 | BPD II | Inpt | citalopram, trazodone, lamictal | 43 | 22 |
| **514** | F | 49 | MDD, OCD | Inpt | None | 47 | 20 |
| **515** | F | 61 | BPD I | Outpt | prozac, zyprexa, neurontin, ativan | 37 | 17 |
| **523** | F | 54 | MDD w/ psychosis, anxiety | Outpt | buspar, tramadol, topamax | 36 | 20 |
| **525** | F | 26 | MDD, anxiety | Outpt | wellbutrin, paxil, ativan | 46 | 20 |
| **527** | F | 48 | MDD | Outpt | wellbutrin, celexa, lamictal | 5^B^ | 11^B^ |
| **528** | F | 60 | BPD I | Inpt | remeron, lithium, lamictal, klonopin | 33 | 19 |
| **530** | F | 49 | BPD II | Outpt | cymbalta, abilify, trileptal | 37 | 19 |
| **532** | F | 28 | MDD, anxiety, PTSD | Outpt | cymbalta, lamictal, xanax | 38 | 25 |
| **535** | F | 42 | BPD I | Outpt | lithium, risperdal, trileptal | 10 | 22 |
| **537** | F | 29 | MDD, anxiety, dysthymia | Outpt | prozac, xanax | 15 | 10 |
| **538** | F | 30 | BPD I, PTSD | Outpt | cymbalta, wellbutrin | 40 | 23 |
| **539** | F | 43 | MDD | Outpt | celexa, wellbutrin | 34 | 21 |
| **541** | F | 55 | BPD I | Outpt | cymbalta | 37 | 18 |
| **542** | F | 72 | BPD II | Outpt | seroquel, neurontin | 31 | 24 |
| **544** | F | 26 | BPD II | Outpt | klonipin, topomax | 29 | 14 |

^A^Only antidepressant, antipsychotics, and anticonvulsants are listed.

^B^Patient suspected of underreporting both HRSD and QIDS
