# Supplementary material for: Dysregulation of leukocyte gene expression in women with medication-refractory depression versus healthy non-depressed controls
Source: BMC Psychiatry. 2013 Oct 21;13:273. doi: 10.1186/1471-244X-13-273 (PMC4015603; doi:10.1186/1471-244X-13-273)
Supplement: Additional file 2: Table S2 — List of ABI Primers (Taqman Assays) used for qPCR. [file 1471-244X-13-273-S2.doc]

**Supplemental Table 2: List of ABI Primers (Taqman Assays) used for qPCR**

| **Gene Symbol** | **ABI numbers** |
| --- | --- |
| TF2B | Hs00155321_m1 |
| APP | Hs01552283_m1 |
| ASIC1 | Hs00241630_m1 |
| ASIC3 | Hs00245097_m1 |
| CREB1 | Hs00231713_m1 |
| DBI | Hs00220950_m1 |
| IL-10 | Hs00174086_m1 |
| IL-6 | Hs00174131_m1 |
| LTA | Hs00236874_m1 |
| NFKB1 | Hs00765730_m1 |
| NR3C1 | Hs01005217_m1 |
| NR3C2 | Hs01031809_m1 |
| NRG1 | Hs00247620_m1 |
| OXT | Hs00792417_g1 |
| OXTR | Hs00168573_m1 |
| P2X1 | Hs00175686_m1 |
| P2X4 | Hs00175706_m1 |
| P2X7 | Hs00175721_m1 |
| P2Y1 | Hs00704965_s1 |
| P2Y2 | Hs04176264_s1 |
| PPARA | Hs00947537_m1 |
| SIRT1 | Hs01009006_m1 |
| SPARC | Hs00234160_m1 |
| STAT5A | Hs00234181_m1 |
| TNF | Hs00174128_m1 |
| TRPV1 | Hs00218912_m1 |
| TRPV4 | Hs01099348_m1 |
| VEGFA | Hs99999070_m1 |
